# Effect of Coffee and Cocoa-Based Confectionery Containing Coffee on Markers of DNA Damage and Lipid Peroxidation Products: Results from a Human Intervention Study

**DOI:** 10.3390/nu13072399

**Published:** 2021-07-13

**Authors:** Daniela Martini, Raúl Domínguez-Perles, Alice Rosi, Michele Tassotti, Donato Angelino, Sonia Medina, Cristian Ricci, Alexandre Guy, Camille Oger, Letizia Gigliotti, Thierry Durand, Mirko Marino, Hans Gottfried-Genieser, Marisa Porrini, Monica Antonini, Alessandra Dei Cas, Riccardo C. Bonadonna, Federico Ferreres, Francesca Scazzina, Furio Brighenti, Patrizia Riso, Cristian Del Bo’, Pedro Mena, Angel Gil-Izquierdo, Daniele Del Rio

**Affiliations:** 1 Department of Food, Environmental and Nutritional Sciences (DeFENS), Università degli Studi di Milano, 20133 Milan, Italy; daniela.martini@unimi.it (D.M.); letizia.gigliotti@unimi.it (L.G.); mirko.marino@unimi.it (M.M.); marisa.porrini@unimi.it (M.P.); patrizia.riso@unimi.it (P.R.); 2 Research Group on Quality, Safety, and Bioactivity of Plant Foods, Department of Food Science and Technology, CEBAS-CSIC, Espinardo, 30100 Murcia, Spain; rdperles@cebas.csic.es (R.D.-P.); smescudero@cebas.csic.es (S.M.); 3 Human Nutrition Unit, Department of Food and Drugs, University of Parma, 43125 Parma, Italy; alice.rosi@unipr.it (A.R.); michele.tassotti@unipr.it (M.T.); francesca.scazzina@unipr.it (F.S.); furio.brighenti@unipr.it (F.B.); daniele.delrio@unipr.it (D.D.R.); 4 Faculty of Bioscience and Technology for Food, Agriculture, and Environment, University of Teramo, 64100 Teramo, Italy; donato.angelino@unite.it; 5 Pediatric Epidemiology, Department of Pediatrics, University Medicine Leipzig, 04103 Leipzig, Germany; Cristian.Ricci@medizin.uni-leipzig.de; 6 Institut des Biomolécules Max Mousseron, IBMM, University of Montpellier, CNRS, ENSCM, 34093 Montpellier, France; alexandre.guy@umontpellier.fr (A.G.); camille.oger@umontpellier.fr (C.O.); thierry.durand@umontpellier.fr (T.D.); 7 BIOLOG Life Science Institute, 28199 Bremen, Germany; hgg@biolog.de; 8 Department of Medicine and Surgery, University of Parma, 43126 Parma, Italy; mantonini@ao.pr.it (M.A.); alessandra.deicas@unipr.it (A.D.C.); riccardo.bonadonna@unipr.it (R.C.B.); 9 Department of Food Technology and Nutrition, Molecular Recognition and Encapsulation (REM) Group, Universidad Católica de Murcia, UCAM, 30107 Murcia, Spain; fferreres@ucam.edu

**Keywords:** coffee, DNA damage, comet assay, lipid peroxidation, oxidative stress, randomized controlled trial

## Abstract

The effect of coffee and cocoa on oxidative damage to macromolecules has been investigated in several studies, often with controversial results. This study aimed to investigate the effect of one-month consumption of different doses of coffee or cocoa-based products containing coffee on markers of DNA damage and lipid peroxidation in young healthy volunteers. Twenty-one volunteers were randomly assigned into a three-arm, crossover, randomized trial. Subjects were assigned to consume one of the three following treatments: one cup of espresso coffee/day (1C), three cups of espresso coffee/day (3C), and one cup of espresso coffee plus two cocoa-based products containing coffee (PC) twice per day for 1 month. At the end of each treatment, blood samples were collected for the analysis of endogenous and H_2_O_2_-induced DNA damage and DNA oxidation catabolites, while urines were used for the analysis of oxylipins. On the whole, four DNA catabolites (cyclic guanosine monophosphate (cGMP), 8-OH-2′-deoxy-guanosine, 8-OH-guanine, and 8-NO2-cGMP) were detected in plasma samples following the one-month intervention. No significant modulation of DNA and lipid damage markers was documented among groups, apart from an effect of time for DNA strand breaks and some markers of lipid peroxidation. In conclusion, the consumption of coffee and cocoa-based confectionery containing coffee was apparently not able to affect oxidative stress markers. More studies are encouraged to better explain the findings obtained and to understand the impact of different dosages of these products on specific target groups.

## 1. Introduction

Coffee is one of the most commonly consumed beverages in the world, and it has always been of great scientific interest because of its potential benefits on health. It is a good source of bioactives, particularly phenolic acids, both free and conjugated with quinic acids (i.e., chlorogenic acids). Coffee is also well known for its content of alkaloids, both purine ones, such as caffeine and theobromine, and pyridine ones, such as trigonelline and its derivatives *N*-methylpiridinium and nicotinic acid [1,2]. The amount and the type of such bioactives in the final beverage depend on several factors, including the blend of coffee (i.e., Arabica or Robusta), the growing area, the extent of roasting, and the coffee brewing, i.e., infusion, moka, or soluble [3,4].

It is postulated that coffee bioactives may play an important role against oxidative stress, a condition caused by an accumulation of reactive oxygen species in cells and tissues. For instance, chlorogenic acids can have a positive effect due to their capacity to increase the endogenous antioxidant defense [5], and caffeine may also play a role, improving protection against free radicals [6,7]. Another important contribution may derive by the action of some compounds that originate during the thermal reactions of the roasting process (i.e., Maillard reaction), such as melanoidins, which have shown potent antioxidant activity [1].

On the whole, the majority of the evidence supporting the role of coffee on oxidative stress derives from in vitro and epidemiological studies. For instance, epidemiological studies have shown an inverse association between coffee consumption and the risk of many chronic non-communicable diseases associated with oxidative stress, such as cardiovascular diseases, diabetes, and several types of cancer [8,9,10]. However, coffee has also been the subject of several intervention trials [11]. Some studies have investigated the capacity of coffee and coffee bioactives to affect reliable markers of oxidative damage to DNA, often estimated through the comet assay, a technique that has been widely used to measure the impact of foods or diet on different markers, such as resistance to oxidatively induced DNA damage and oxidized basis [12,13,14]. Despite some studies finding a beneficial effect of coffee on these markers [15,16], others showed no significant effect [17,18]. Similarly, some studies have also evaluated the role of coffee on oxidative stress to lipids (e.g., isoprostanes, oxidized low-density lipoproteins, or malondialdehyde) and proteins (e.g., protein carbonyls) [1,5,19,20]. However, findings were often inconclusive also because of the high heterogeneity among the studies [19]. For instance, while some reported a reduction on lipid peroxidation markers following coffee consumption [21,22], other studies found no effect on these markers [17,23].

In light of these controversial observations, there is a clear need to perform dietary intervention studies devoted to elucidating the impact of coffee consumption on human health. Within this context, the Pocket-4-life project [24] was developed with the specific purpose of investigating the bioavailability of the bioactive compounds of espresso coffee and cocoa-based confectionery containing coffee [25,26], as well as to study the potential beneficial effect of these products on cardiometabolic and oxidative stress markers [24,27]. Specifically, within the Pocket-4-Life project, we investigated the effect of one-month consumption of different doses of coffee or of cocoa-based products containing coffee on markers of oxidative stress, such as DNA damage and lipid peroxidation. The addition of cocoa-based products to the study was linked to the emerging evidence that cocoa and related products are also rich in antioxidants, such as flavonoids, proanthocyanidins, and phenolic acids, with a significant impact on the reduction of some markers of oxidative stress, for example, markers of lipid peroxidation, such as malondialdehyde (MDA) and 8-iso-prostaglandin F2α (15-F_2t_-Isoprostane), which can play an important role in the modulation of cardiovascular health [5,28].

## 2. Materials and Methods

### 2.1. Products

Subjects received a single-serve coffee machine (Essenza EN 97.W, De’Longhi Appliances S.p.a., Treviso, Italy), coffee capsules (Capriccio, Nespresso Italia S.p.a., Assago, Italy), and cocoa-based products containing coffee (Pocket Coffee, Ferrero Commerciale Italia S.r.l., Alba, Italy).

As already reported [25,26,27], coffee capsules were chosen based on the results of a previous paper [2], while the amount of cocoa-based confectionery containing coffee was decided in order to provide approximately half of the caffeine content than the coffee.

### 2.2. Participants

A group of 21 healthy volunteers was enrolled in Parma (North Italy) using announcements placed in university, hospital, and public places. Subjects received all the information and details about the protocol and the potential risks associated with participation, and they signed a written consent form.

Inclusion criteria included being adult (>18 years old), healthy, normal weight (body mass index (BMI) 18–25 kg/m^2^), and habitual consumers of 1 to 5 cups of espresso coffee per day. Exclusion criteria included clinical diagnosis for metabolic, renal, or digestive disorders; regular consumption of medication; antibiotic therapy taken within the last 3 months; intense physical activity; pregnancy or lactation; and habitual very high consumption of coffee/cocoa-related phytochemicals (e.g., more than 5 coffees/day). These criteria were set to avoid likely confounding factors.

### 2.3. Study Design and Protocol

The study protocol received approval by the Ethics Committee of the University of Parma (AZOSPR/0015693/6.2.2.) and was registered at clinicaltrials.gov on May 21, 2017 (as NCT03166540).

The study design has already been published elsewhere [24]. Briefly, in a three-arm, crossover trial, 21 subjects were randomly assigned to receive 3 different treatments in a random order for 1 month: 1 cup of espresso coffee/day (from now on named “1C group”), 3 cups of espresso coffee/day (from now on “3C group”), and 1 cup of espresso coffee plus 2 cocoa-based products containing coffee twice per day (from now on “PC group”). Random Number Generator Pro (Segobit Software) was used to generate a randomization list.

During the three interventions, volunteers maintained their usual dietary habits and were instructed to avoid other coffee/cocoa-related food sources for the 2 days prior to and during the sampling day. To this aim, a list of allowed and prohibited foods was provided to the volunteers as previously reported [27].

On the sampling day, the subjects referred in the morning at the ambulatory of the Endocrinology Unit of the Department of Medicine and Surgery of the University of Parma for the visits. Fasting baseline urine and blood samples were collected, then subjects received one of the three treatments depending on the randomization. Briefly, 1C and 3C subjects consumed one (at 9.00 a.m.) or three cups of espresso coffee (at 9.00 a.m., 12.00 p.m., and 3.00 p.m.), respectively, while PC subjects consumed one cup of espresso coffee (at 9.00 a.m.) and 2 cocoa-based products containing coffee twice during the day (at 12.00 p.m. and 3.00 p.m.). One-month after the beginning of each treatment, subjects were asked to return to the ambulatory and both fasting urine and blood samples were collected.

### 2.4. Total Antioxidant Capacity of the Diet

During the enrollment, subjects were asked to fill in a 53-item semi-quantitative food frequency questionnaire (FFQ) for the assessment of dietary total antioxidant capacity (TAC) [29]. TAC was expressed as Trolox equivalent antioxidant capacity (TEAC), total radical-trapping antioxidant parameter (TRAP), and ferric reducing antioxidant power (FRAP).

### 2.5. Analysis of Markers of DNA Damage by Comet Assay

#### 2.5.1. Chemicals and Reagents

All chemicals and reagents used for the analysis of DNA damage were obtained from Merck (Darmstadt, Germany). GelBond^®^ films were from VWR International S.r.l. (Monroeville, PA, USA), while the formamidopyrimidine DNA glycosylase (FPG) enzyme was obtained by Norgenotech AS (Oslo, Norway).

#### 2.5.2. Sample Preparation and Analysis

Blood was obtained from an intravenous catheter, and samples were collected into tubes containing heparin as an anticoagulant. Peripheral blood mononuclear cells (PBMCs) were obtained by density gradient centrifugation with Histopaque^®^ 1077, washed with phosphate-buffered saline (PBS), resuspended in a freezing media, and stored at −80 °C until analysis, as previously reported [30,31].

The levels of H_2_O_2_-induced DNA damage (i.e., oxidatively induced DNA damage) and FPG-sensitive sites (i.e., endogenous oxidative base damage) were analyzed in PBMCs by the comet assay. The full protocols have been previously published [30,31,32].

### 2.6. Analysis of DNA Oxidation Catabolites

#### 2.6.1. Chemicals and Reagents

The cyclic guanosine monophosphate (cGMP), 8-NO2-guanine, 8-OH-guanine, 8-NO2-cGMP, and 8-NO2-guanosine were from the Biolog Life Science Institute (Bremen, Germany). The 8-OH-2′-deoxy-guanosine and 8-OH-guanosine were from Cayman Chemicals (Ann Arbor, MI, USA). Solvents used for the liquid chromatography coupled to mass spectrometry analyses were obtained from J.T. Baker (Phillipsburg, NJ, USA), and the deionized water was supplied by a Millipore system. Acetic acid, sodium hydroxide, and ammonium acetate were purchased from Panreac (Castelar del Vallés, Barcelona, Spain).

#### 2.6.2. Extraction and Processing of the Samples

The solid phase extraction (SPE) of DNA catabolites was carried out with ISOLUTE (Env, 1 mL, 50 mg) cartridges (Biotage, Tokyo, Japan), according to the methodology previously described [33,34].

#### 2.6.3. Analysis of DNA Oxidation Catabolites

Chromatographic analyses were carried out with a UHPLC coupled to a 6460 QqQ-MS/MS (Agilent Technologies, Waldbronn, Germany) equipped with an electrospray ionization (ESI) source, following the analytical methodology previously reported [33,34,35].

### 2.7. Analysis of Lipid Oxidation Catabolites (Oxylipins)

#### 2.7.1. Chemicals and Reagents

HPLC-grade ACN and methanol were obtained from Scharlau Chemie (Barcelona, Spain). Ultrapure water (Milli-Q) was from Milli-Q Gradient A10 system (Millipore, Bedford, MA, USA). β-glucuronidase, type H2 from *Helix pomatia*, and BIS-TRIS (Bis-(2-hydroxyethyl)-amino-tris(hydroxymethyl)-methane) were from Sigma-Aldrich (St. Louis, MO, USA). Strata X-AW cartridges, 100 mg 3 mL-1, were from Phenomenex (Torrance, CA, USA). The authentic standards used for quantification (15-F_2t_-IsoP, *ent*-15-*epi*-15-F*2t*-IsoP, 2,3-dinor-15-F_2t_-IsoP, 2,3-dinor-15-*epi*-15-F_2t_-IsoP, 5-F_2t_-IsoP, 5-*epi*-5-F_2t_-IsoP) were synthesized following the methodology previously described [36,37,38]. The additional IsoP and prostaglandin (PG) standards were obtained from Cayman Chemical (Ann Arbor, MI, USA).

#### 2.7.2. Extraction and Processing of the Samples

The extraction of human oxylipins in urine, the pre-processing with analytical purposes, and the UHPLC-QqQ-MS/MS-based analyses were carried out following methods previously developed [39,40,41,42,43].

#### 2.7.3. Analysis of the Lipid Oxidation Catabolites

LC-MS/MS has been demonstrated to be the most specific and versatile method to determine biomarkers of lipid peroxidation [44,45,46,47]. The urinary concentration of the eicosanoids was calculated from the area ratio of the ion peaks of the compounds over the corresponding authentic standards.

### 2.8. Statistical Analysis

Sample size was calculated by considering the daily average concentration of a coffee-derived plasma circulating phenolic metabolites as the primary outcome, as already reported [24]. In detail, 15 subjects were necessary to detect a change of 600 nmol/h/L^−1^ in dihydrocaffeic acid-3′-sulfate plasma concentration with a standard deviation (SD) of 870 nmol/h/L^−1^ (α error of 0.05, 80% power, and 2-sided testing). The sample size was also preliminarily estimated as appropriate for all the secondary outcomes since it reflected the sample size considered in other trials that evaluated the effects of coffee intake on the same markers. Furthermore, a post hoc power calculation was conducted for secondary outcomes. Specifically, considering a type-I error rate of 5%, a type-II error rate of 20% (power 80%), and 21 subjects, we calculated that a medium-to-large, standardized effect size (Cohen’s d) could be detected being in the measure of 0.89 and 0.64 for the two samples and paired t-test, respectively.

Data are presented as mean ± SEM. A generalized linear model for repeated measurements was performed to test the effect of time and treatment (within-subjects factor) on markers of DNA damage (dependent variables), also considering sex, smoking habits, TEAC, FRAP, and TRAP (between-subject factors). Violation of the assumptions of normality, homogeneity of variances, and the reliable measurement of the covariates were checked. In addition, if a main effect of treatment was registered, Bonferroni post hoc tests were used for multiple comparisons. Carry-over effects were firstly investigated comparing the effects given by two opposite treatment sequences over the same outcome. Afterward, a Wald test on the coefficient of the interaction term between treatment and time was performed [48].

A difference was considered significant at *p* < 0.05. The statistical analysis was performed with the Statistical Package for Social Sciences software (IBM SPSS Statistics for Macintosh, Version 26.0. Armonk, NY: IBM Corp). Power calculations were performed using the pwr package of the R software (version 3.6.3) with type-I and type-II error rates of 5% and 20%, respectively.

## 3. Results

### 3.1. Characteristics of Subjects

All the enrolled participants (*n* = 21; 11 females) completed the study. No side effects were reported in any of the subjects. The main characteristics of the subjects at baseline are reported in Table 1. Interestingly, smokers showed high levels of DNA strand breaks, but not of FPG-sensitive sites and H_2_O_2_-induced DNA damage, compared to non-smokers (29.1 ± 2.1% vs. 23.2 ± 1.6%, *p* = 0.036, respectively; DNA strand breaks in PBS). No differences were observed for the variable sex nor for dietary total antioxidant capacity (data not shown).

### 3.2. Markers of DNA Damage

#### 3.2.1. Endogenous and Oxidatively Induced DNA Damage

Table 2 reports the effect of different coffee dosages and of its partial substitution with cocoa-based products containing coffee on markers of DNA damage as evaluated by the comet assay. As shown, no effects were reported for the levels of H_2_O_2_-induced DNA damage nor for FPG-sensitive sites in any of the three intervention arms. No effect was reported comparing pre- to post-intervention. Conversely, a significant effect of time, but not of the treatment, was reported for the levels of DNA strand breaks showing a significant reduction following intervention with 3C and PC. When the model was adjusted for covariates (sex, smoking habits, TEAC, FRAP, and TRAP), interactions revealed no significant main effects for the considered outcomes. No carry-over effects were observed (Table S1).

#### 3.2.2. DNA Oxidation Catabolites

Table 3 shows the effect of the three treatments on three DNA oxidation catabolites and cGMP in human plasma of the volunteers. Four DNA catabolites were detected in the plasma samples of the volunteers, i.e., cGMP, 8-OH-2′-deoxy-guanosine, 8-OH-guanine, and 8-NO2-cGMP. The other three catabolites (8-OH-guanosine, 8-NO2-Guo, and 8-NO2-Gua) were not detected in any of the plasma samples. No effects were detected in cGMP and the three DNA oxidation catabolites and cGMP after any of the three nutritional interventions. Carry-over effects were not shown (Table S2).

### 3.3. Lipid Peroxidation Products (Oxylipins)

Table 4 shows the total quantitative variations of lipid oxidation catabolites evaluated pre- to post-intervention and a comparison of the three treatments. A significant time reduction in total prostaglandins F-pathway was observed following 3C and PC, while a significant increase in total F2-isoprostanes 5 series was reported only following treatment with 3C. Lower values, but not statistically significant, were detected for total E_2_-isoprostanes 15-series, total F_2_-isoprostanes 15 series, total prostaglandins F-pathway, and total IsoPs from dihomo-γ-linolenic acid upon cocoa-based products containing coffee twice per day administration (PC) compared to 1C and 3C (treatment effect).

The analysis of the individual IsoPs and PGs from arachidonic acid and dihomo-γ-linolenic acid confirmed the previous results obtained for the total quantitative values of these compounds (Table 5). These individual oxylipins remained unchanged regardless of the three intervention arms (1C, 3C, and PC), while a significant effect of time (pre- to post-intervention) was observed for some of the markers analyzed (Table 5). No carry-over effects were observed (Table S3).

## 4. Discussion

We evaluated through a three-arm, randomized, crossover study the effects of a one-month intervention with coffee and cocoa-based products containing coffee on markers of DNA damage and lipid peroxidation (oxylipins) in a group of healthy subjects. To the best of our knowledge, this is the first trial combining and investigating the effects of coffee and cocoa on such a broad panel of these specific markers. Despite the numerous in vitro and epidemiological evidence reporting positive associations between coffee and cocoa consumption and several health indicators [10,49], in our experimental conditions, there are no differences among the effects of consuming one or three espresso coffees per day for one month and of their partial substitution with confectionary containing coffee on the markers associated with DNA degradation, oxidative stress, and inflammation-based oxylipins. Specifically, the intake of coffee at different dosages had no effect on endogenous and oxidatively induced DNA damage (evaluated as FPG-sensitive sites and resistance to H_2_O_2_ oxidative insult, respectively), as markers of oxidative stress, in healthy volunteers. These results could be partially explained by the use of cryopreserved cells. In fact, we have previously documented that, while FPG-sensitive sites can be considered as a reliable biomarker for the assessment of DNA damage, both in cryopreserved and fresh samples, H_2_O_2_ as oxidative insult in cryopreserved cells is widely used even if debated [32,50]. Based on these premises, we cannot exclude that the freezing process and the time of storage could have affected the cell response to induced DNA oxidative stress in our experimental conditions. This point deserves further investigation, and it could represent a possible limitation of the study.

The effect of coffee and coffee-based products on these markers has been evaluated in other several human intervention studies both in fresh as well as in cryopreserved cells, and the results are still unclear. Shaposhnikov and colleagues [17] reported that an eight-week intervention with three or five cups of coffee per day failed to affect the levels of base oxidation in the DNA of PBMCs in a group of healthy volunteers. Hoelzl and colleagues [18] showed that the intake of 800 mL/day of instant coffee (co-extracted from green and roasted beans) for 5 days did not significantly reduce DNA damage, evaluated as FPG-sensitive sites and H_2_O_2_-induced DNA damage, in healthy individuals. Misik et al. [51], in a crossover study, demonstrated that the daily consumption of 800 mL of coffee over 5 days failed to reduce the levels of H_2_O_2_-induced DNA damage, while it decreased the levels of oxidized purines (FPG-sensitive sites) in healthy subjects. Conversely, other studies reported a beneficial effect of coffee intake, as Steinkellener and colleagues [15] reported an increase in cell DNA resistance to oxidative damage following a five-day consumption of 1 L of coffee. Similar observations were reported by Bichler et al. [16] after the consumption of 600 mL of coffee (400 mL paper filtered and 200 mL metal filtered/day) for five days in healthy volunteers. The authors documented a decrease in the levels of purines and pyrimidines in lymphocyte DNA (detected by using FPG and EndoIII, respectively) as well as on oxidatively induced DNA damage, measured as DNA resistance to both H_2_O_2_ and Trp-P-2. Cardin and colleagues observed a positive effect on 8-OH-2′-deoxy-guanosine in patients with chronic hepatitis C following the consumption of four cups of coffee/day for 30 days [52]. More recently, Pahlke et al. [53] showed that an eight-week intervention with 750 mL of coffee blend reduced spontaneous DNA strand breaks in healthy subjects, and Scipp and colleagues [54] demonstrated that 500 mL of freshly brewed dark roast coffee blend per day for 4 weeks reduced the levels of DNA strand breaks in healthy volunteers. Similar results were obtained by Bakuradze and colleagues, who found a decrease in endogenous and oxidatively induced DNA damage following 4 weeks consumption of 750 mL of freshly brewed black filtered coffee [55], dark coffee [56], and dark roast coffee [57] in healthy individuals. In addition, the same authors found a decrease in background DNA strand breaks in healthy male subjects following a short-term intervention with 800 mL of coffee (i.e., 200 mL of black coffee aliquots every second hour up to 8 h) [58].

Regarding DNA strand breaks, we documented high levels of DNA damage at baseline in smokers compared to non-smokers, in line with the observations of different meta-analyses [59,60]. On the other hand, the differences observed were not attributed to differences in their diet as documented by comparable values in terms of dietary total antioxidant capacity and dietary habits [27].

When comparing the effect of coffee and coffee-based products, we found a reduction in DNA strand breaks following the intervention, in line with other authors [53,54,55,56,57,58]. Since the reduction was only observed following the 3C and PC treatments, but not 1C, we may hypothesize the potential contribution of the dosage (3C) and of the mix of the products (PC) in the modulation of DNA damage. However, it is worth noting that the reduction observed was attributed to an effect of time but not of the treatment.

To the best of our knowledge, the effects of cocoa and cocoa-derived products on DNA protection have been evaluated only in two studies and never in combination with coffee. Spadafranca and coworkers [61] showed that dark chocolate increased DNA resistance to oxidative insult induced by H_2_O_2_, but only after 2 h of ingestion dark chocolate vs. white chocolate, while no effect has been documented after 2 weeks in normal weight young healthy subjects. This protection was correlated with the amount of epicatechin in plasma. More recently, Ibero-Baraibar and colleagues reported no effect on endogenous strand breaks, oxidized bases, and resistance to H_2_O_2_-induced damage after 4 weeks of intervention in a group of middle-aged overweight/obese subjects consuming ready-to-eat meals supplemented with 1.4 g of cocoa extract in the context of a hypocaloric diet [62].

Regarding the specific individual markers responsible for a reflection of the protective effect of coffee or cocoa-based products, it should be detailed that all the DNA oxidation markers analyzed were derived from the nucleotides and nucleosides of DNA. Nevertheless, their potential biological action and utilization as pathogenic markers are strictly dependent on the individual compound considered. While the raise of cGMP level in the plasma could reflect a decrease in vascular contraction (marker of vasodilation), increased plasma 8-OH-2′-deoxy-guanosine, 8-OH-guanine, and 8-NO_2_-cGMP represent an accurate reflection of increased oxidative stress [34]. To the best of our knowledge, there are no previous reports describing the behavior of these DNA oxidation catabolites in humans after coffee or cocoa-based product consumption. However, a positive reduction of these markers has been observed after the intake of other polyphenol-rich plant-based drinks, such as wine or citrus juices [34,35].

The impact of coffee and cocoa on lipid damage and related markers has been evaluated in different studies. A review summarized the effects of coffee on different markers of oxidative stress, including lipid peroxidation, and concluded that the effects are controversial [19]. Overall, in our experimental conditions, the consumption of coffee and cocoa-based confectionery containing coffee did not affect markers of lipid peroxidation, apart from a significant increase in total F2-isoprostanes 5 series following treatment with 3C and reduction in total prostaglandins F-pathway following 3C and PC, in line with the findings on DNA strand breaks, thus suggesting again a possible contribution of the dose and of the mix of the products (coffee and cocoa-based products) in the modulation of the markers. However, these findings were attributed to a time effect, while no significant differences were reported comparing the three treatments. Our findings are consistent with observations reported by other researchers after acute [63,64,65] and chronic [51,63,66] ingestion of coffee. For example, Marsu and coworkers [23] found no effect of a three-week intervention with up to six cups of filtered coffee per day on plasma IsoPs and conjugated dienes in LDL cholesterol, as markers of lipid peroxidation, in healthy subjects. Shaposhnikov and colleagues [17] reported that 8 weeks of intervention with three or five cups of coffee or water per day failed to affect urine IsoP levels in a group of healthy volunteers. Conversely, other studies reported a reduction on lipid peroxidation markers, as Kempf et al. [21] showed that the intake of eight cups (150 mL/cup) of filtered coffee/day compared to zero and four cups/day reduced the levels of serum 8-IsoP following a one-month intervention in subjects with an elevated risk of type-2 diabetes. Yakawa et al. [22] found that subjects drinking 150 mL of coffee three times per day for one week had reduced plasma levels of MDA, a marker of lipid peroxidation, in healthy male students. Finally, Hoelzl et al. [18] reported a significant reduction in urinary 15-F_2t_-IsoP, but not plasma Ox-LDL and MDA, following the consumption of instant coffee. Recently, a nutritional intervention with coffee with high content of chlorogenic acids versus normal content of chlorogenic acids showed a reduction of human oxylipins, including different types of IsoPs and prostaglandins, after both types of coffees [5]. Conversely, cocoa and cocoa-based products have shown to counteract lipid peroxidation, in terms of MDA and 8-iso-prostaglandin F2α (15-F_2t_-IsoP) levels, as documented by a recent systematic review and meta-analysis of intervention studies [28].

The variability observed among studies can be attributed to several important factors, including the different food matrix provided to the volunteers, the different (poly)phenol content characterizing coffee and cocoa products, and the fact that these products have never been investigated in combination. Furthermore, when two types of coffee with different processing treatments resulting in very different chlorogenic acid content were fed to volunteers, the one with the higher content exerted a more relevant decrease of 12 human oxylipins (IsoPs and PGs) [5]. Another factor can be ascribable to the different durations of the studies. In this regard, in the present trial, we investigated the effect of 1 month of coffee consumption, similarly to previous studies of 4 weeks per arm [52,54,57,58], but studies evaluating the effect of coffee consumed for just a few days (i.e., 3–5 days) [15,16,18,51] are also present in the literature. In this regard, it has been speculated that long-term treatments might trigger a homeostatic response of the organism, which restores the initial conditions for some of the tested variables; thus, the lack of a modulation observed in our markers might be attributed to this form of adaptation. However, since we missed early timepoints during each treatment, we are not able to infer whether it really is the effect of an adaptive homeostasis. Another, paramount, factor is certainly the amount of coffee, as most of the reported studies [15,18,51,55,57,58] provided higher coffee volumes (i.e., up to 1 L/day) compared to the 40 mL/day and 120 mL/day provided by one and three espresso coffees in our realistic experimental conditions. Despite the fact that the amount of coffee is not necessarily a proxy of the amount of bioactives, we cannot exclude that the different results obtained can be partially attributed to the low dosage of coffee provided to the volunteers. The variability in terms of caffeine and phenolic compounds (chlorogenic acids in particular) among studies may partially explain the differences observed, as the content of these bioactives may depend on coffee variety, roasting, and brewing method [4]. In our study, one and three coffees per day provided on average 74 mg and 222 mg of caffeine, respectively, and 73 mg and 219 mg of chlorogenic acids, respectively, while studies present in the literature provided in some cases a much higher amount of caffeine, with Pahlke et al. [53] providing up to 300 mg of caffeine/day and Bakuradze and coworkers more than 500 mg of caffeine/day [55]. Lara-Guzmán et al. [5] provided coffee-based dosages of chlorogenic acids per day of 787 mg and 407 mg, depending on the type of processing. However, the comparison among studies is difficult, not only for the different composition of the various types of coffee but also for the missing data on caffeine content and in phenolic compounds for many studies. Moreover, it is noteworthy that a comparison with previous findings is further complicated because some studies compared the effects of coffee consumption with no coffee, while in the present study, we investigated the impact of different dosages.

Finally, our study was conducted in free-living subjects who kept their lifestyle unchanged during the three intervention periods, also in terms of consumption of (poly)phenol-rich foods, with the only exception for the tested products and for a small higher energy intake in subjects consuming the cocoa-based products [27]. This represents a strength of the trial because it mimics a realistic condition, but it may also represent a source of variation compared to previous investigations in which subjects consumed a low-(poly)phenol diet. Another difference compared to other studies regards the lack of a run-in and wash-out period in order to maintain the free-living conditions. This experimental approach may partially explain the results obtained. A further explanation of the lack of significant findings could be related to the high inter-individual variability in the response to the treatments, as already observed [67,68].

## 5. Conclusions

In conclusion, the results obtained in this intervention study showed no differences among the effect of different dosages of espresso coffee and their partial substitution with cocoa confectionary containing coffee on markers of oxidative stress in young healthy volunteers. These findings are in agreement with those on cardiometabolic risk factors, obtained within the same trial [27], but in contrast with other studies supporting the potential beneficial role of coffee and products containing coffee [19,69,70]. Thus, further studies should be performed to corroborate our findings and to evaluate the impact of coffee and chocolate, alone and/or in combination, in different target groups (e.g., subjects with risk factors) and on a wider panel of oxidative stress markers.

## Figures and Tables

**Table 1:** Characteristics of subjects at baseline.

Variable	All Participants (*n* = 21)
*Socio-demographic and anthropometric data*	
Age (y)	22.9 ± 0.5
Sex (males/females)	10/11
Smoking habits (smokers/non-smokers)	8/13
Body weight (kg)	67.0 ± 2.7
Body mass index (kg/m^2^)	22.3 ± 1.7
Habitual coffee consumption (serving/day)	2.3 ± 0.2
*Dietary Total Antioxidants Capacity*	
TAC (mmol Trolox eq./day)	8.2 ± 3.3
TRAP (mmol Trolox eq./day)	11.8 ± 5.6
FRAP (mmol Fe^2+^ eq./day)	26.1 ± 11.2
*Markers of DNA damage*	
DNA strand breaks (% DNA in tail, PBS)	25.4 ± 1.6
H_2_O_2_-induced DNA damage (% DNA in tail)	14.4 ± 1.5
DNA strand breaks (% DNA in tail, EB)	27.4 ± 2.1
FPG-sensitive sites (% DNA in tail)	10.3 ± 1.2
8-OH-guanine (nM)	25.3 ± 5.2
8-NO_2_-cGMP(nM)	58.1 ± 13.9
8-OH-2′-deoxy-guanosine (nM)	17.4 ± 2.3
cGMP (nM)	96.3 ± 4.9
*Markers of lipid peroxidation*	
**Oxylipins from Arachidonic Acid**	
**PGs**	
**D-Pathway**	
2,3-dinor-11*β*-PGF_2_ (µM)	2.50 ± 0.24
11-β-PGF2_α_ (µM)	0.09 ± 0.01
Tetranor PGDM (µM)	0.40 ± 0.02
PGDM (µM)	0.45 ± 0.01
Tetranor PGJM (µM)	0 ± 0
Tetranor PGDM lactone (µM)	0 ± 0
**E-Pathway**	
Tetranor PGAM (µM)	6.38 ± 2.21
Tetranor PGEM (µM)	0.28 ± 0.02
20-OH-PGE_2_ (µM)	0.27 ± 0.03
PGE_2_ (µM)	0 ± 0
**F-Pathway**	
Tetranor PGFM (µM)	0.21 ± 0.04
PGF_2α_ (µM)	0.16 ± 0.03
20-OH-PGF_2α_ (µM)	3.47 ± 0.32
19(R)-OH-PGF_2α_ (µM)	0 ± 0
**I-Pathway**	
6-keto-PGF_1α_ (µM)	0 ± 0
**F_2_-IsoPs**	
**15 series**	
2,3-dinor-15-F_2t_-IsoP (2,3-dinor-8-iso-PGF_2α_) (µM)	0.90 ± 0.01
15-*epi*-15-F_2t_-IsoP (8-iso-15(R)-PGF_2α_) (µM)	1.04 ± 0.05
15-F_2t_-IsoP (8-iso-PGF_2α_) (µM)	0.09 ± 0.01
9-*epi*-15-F_2t_-IsoP (8-iso-PGF_2β_) (µM)	0.02 ± 0.00
15-*keto*-15-F_2t_-IsoP (8-iso-15-keto-PGF_2α_) (µM)	0.96 ± 0.00
*ent*-15-*epi*-15-F_2t_-IsoP (Ent-8-iso-15S-PGF_2α_) (µM)	0.61 ± 0.02
2,3-dinor-15-*epi*-15F_2t_ (µM)	1.51 ± 0.27
**5 series**	
5-F_2t_ (µM)	5.89 ± 0.73
5-*epi*-5F_2t_ (µM)	1.32 ± 0.08
**E_2_-IsoPs**	
**15 series**	
15-*keto*-15-E_2t_-IsoP (8-iso-15keto-PGE_2_) (µM)	1.03 ± 0.11
**Oxylipins from Dihomo-γ-linolenic acid**	
PGs	
PGE_1_ (µM)	0.54 ± 0.03
PGD_2_ (µM)	2.02 ± 0.01
PGF_1α_ (µM)	0.48 ± 0.04
**IsoPs**	
15-F_1t_-IsoP (8-iso-PGF_1α_) (µM)	0.003 ± 0.000
15-E_1t_-IsoP (8-iso-PGE_1_) (µM)	4.84 ± 0.68

Data are expressed as mean ± SEM. Legend: FPG: DNA glycosylase; FRAP: ferric reducing antioxidant power; IsoPs: isoprostanes; PGs: prostaglandins; TAC: total antioxidant capacity; TEAC: Trolox equivalent antioxidant capacity; TRAP: total radical-trapping antioxidant parameter.

**Table 2:** Markers of DNA damage after each treatment (*n* = 21).

Variable	1C			3C			PC			*p*-Value ^#^
	Pre	Post	*p*-Value *	Pre	Post	*p*-Value *	Pre	Post	*p*-Value *	
DNA strand breaks (% DNA in tail, PBS)	17.2 ± 1.2	15.5 ± 1.0	0.331	19.8 ± 1.6	18.3 ± 0.9	0.017	20.3 ± 1.3	19.6 ± 0.8	0.005	0.349
H_2_O_2_-induced DNA damage (% DNA in tail)	12.4 ± 0.8	9.8 ± 0.8	0.155	12.6 ± 1.2	11.2 ± 1.3	0.410	11.9 ± 1.3	12.7 ± 1.4	0.678	0.938
DNA strand breaks (% DNA in tail, EB)	20.5 ± 1.6	17.0 ± 1.0	0.076	20.8 ± 1.0	17.7 ± 1.0	0.147	21.2 ± 1.9	17.7 ± 1.0	0.106	0.988
FPG-sensitive sites (% DNA in tail)	7.8 ± 1.3	5.8 ± 0.7	0.178	7.2 ± 0.9	6.9 ± 1.1	0.858	7.6 ± 1.1	6.8 ± 1.0	0.622	0.423

All values are reported as mean ± SEM. Legend: 1C: group consuming 1 cup of espresso coffee/day; 3C: group consuming 3 cups of espresso coffee/day; PBS: phosphate buffer saline; EB: endonuclease buffer; FPG: formamidopyrimidine DNA glycosylase; PC: group consuming 1 cup of espresso coffee plus 2 cocoa-based products containing coffee twice per day. * Effect of time (pre- to post-intervention for each group). Data with *p* < 0.05 are significantly different. ^#^ Effect of treatment (1C vs. 3C vs. PC). Data with *p* < 0.05 are significantly different.

**Table 3:** Markers of DNA oxidation catabolites in plasma after each treatment (*n* = 21).

Variable	1C			3C			PC			*p*-Value ^#^
	Pre	Post	*p*-Value *	Pre	Post	*p*-Value *	Pre	Post	*p*-Value *	
8-OH-guanine (nM)	19.0 ± 5.5	8.7 ± 3.7	0.130	16.2 ± 4.2	9.2 ± 2.7	0.168	10.7 ± 2.6	12.7 ± 3.5	0.642	0.692
8-NO2-cGMP(nM)	55.2 ± 16.9	22.4 ± 13.3	0.136	37.3 ± 14.2	56.4 ± 21.7	0.467	47.1 ± 21.9	48.2 ± 18.6	0.971	0.413
8-OH-2′-deoxy-guanosine (nM)	12.3 ± 2.6	11.0 ± 2.6	0.715	11.1 ± 2.6	13.2 ± 2.6	0.559	14.4 ± 2.5	12.2 ± 2.6	0.545	0.812
cGMP (nM)	101.6 ± 8.0	131.7 ± 35.6	0.415	94.9 ± 6.6	114.2 ± 8.6	0.082	132.2 ± 35.5	107.0 ± 5.6	0.487	0.583

All values are reported as mean ± SEM Legend: 1C: group consuming 1 cup of espresso coffee/day; 3C: group consuming 3 cups of espresso coffee/day; PC: group consuming 1 cup of espresso coffee plus 2 cocoa-based products containing coffee twice per day. * Effect of time (pre- to post-intervention for each group). Data with *p* < 0.05 are significantly different. ^#^ Effect of treatment (1C vs. 3C vs. PC). Data with *p* < 0.05 are significantly different.

**Table 4:** Total markers of lipid oxidation catabolites ordered by metabolic pathway series after each treatment (*n* = 21).

Series	1C			3C			PC			*p*-Value ^#^
	Pre	Post	*p*-Value *	Pre	Post	*p*-Value *	Pre	Post	*p*-Value *	
E_2_-IsoPs 15-series	0.84 ± 0.09	0.77 ± 0.07	0.546	0.79 ± 0.07	0.78 ± 0.08	0.959	0.84 ± 0.03	0.67 ± 0.03	0.116	0.395
F_2_-IsoPs 15 series	4.48 ± 0.32	4.93 ± 0.38	0.374	4.56 ± 0.32	4.75 ± 0.22	0.613	4.63 ± 0.27	4.73 ± 0.27	0.796	0.865
F_2_-IsoPs 5 series	8.35 ± 0.66	10.64 ± 1.26	0.116	7.64 ± 0.78	10.37 ± 0.86	0.023	8.28 ± 0.79	9.68 ± 0.78	0.215	0.767
PGs D-Pathway	4.77 ± 0.22	4.59 ± 0.16	0.507	4.86 ± 0.21	4.54 ± 0.12	0.190	4.62 ± 0.17	4.61 ± 0.20	0.987	0.946
PGs E-Pathway	5.36 ± 2.04	5.13 ± 2.44	0.943	3.90 ± 1.54	7.53 ± 2.82	0.266	8.78 ± 2.85	7.01 ± 3.86	0.716	0.780
PGs F-Pathway	1.26 ± 0.34	0.67 ± 0.19	0.142	1.67 ± 0.37	0.76 ± 0.18	0.034	1.94 ± 0.43	0.52 ± 0.15	0.003	0.669
PGs I-Pathway	nd	nd	-	nd	nd	-	nd	nd	-	-
IsoPs from DGLA	3.37 ± 0.59	3.10 ± 0.46	0.715	3.12 ± 0.47	3.14 ± 0.53	0.978	3.53 ± 0.67	2.40 ± 0.20	0.116	0.395
PGs from DGLA	2.90 ± 0.15	3.08 ± 0.04	0.248	2.88 ± 0.15	3.09 ± 0.05	0.182	3.08 ± 0.04	3.08 ± 0.04	0.921	0.812

All values are expressed as µM and are reported as mean ± SEM. Legend: 1C: group consuming 1 cup of espresso coffee/day; 3C: group consuming 3 cups of espresso coffee/day; PC: group consuming 1 cup of espresso coffee plus 2 cocoa-based products containing coffee twice per day. All values are reported as mean ± SEM; nd: not detected. E2-IsoPs 15-series: total E2-isoprostanes 15-series; F2-IsoPs 15 series: total F2-isoprostanes 15 series; F2-IsoPs 5 series: total F2-isoprostanes 5 series; PGs D-Pathway: total prostaglandins D-pathway; PGs E-Pathway: total prostaglandins E-pathway; PGs F-Pathway: total prostaglandins F-pathway; PGs I-Pathway: total prostaglandins I-pathway; IsoPs from DGLA: total isoprostanes from dihomo-γ-linolenic acid; PGs from DGLA: prostaglandins from dihomo-γ-linolenic acid. * Effect of time (pre- to post-intervention for each group). Data with *p* < 0.05 are significantly different. ^#^ Effect of treatment (1C vs. 3C vs. PC). Data with *p* < 0.05 are significantly different.

**Table 5:** Markers of lipid oxidation catabolites after each treatment (*n* = 21).

Variable	1C			3C			PC			*p*-Value ^#^
	Pre	Post	*p*-Value *	Pre	Post	*p*-Value *	Pre	Post	*p*-Value *	
**Oxylipins from Arachidonic Acid**										
**PGs**										
**D-Pathway**										
2,3-dinor-11*β*-PGF_2_	1.70 ± 0.23	1.59 ± 0.13	0.668	1.84 ± 0.22	1.57 ± 0.10	0.275	1.65 ± 0.15	1.65 ± 0.18	0.977	0.898
11-*β*-PGF2_α_	0.12 ± 0.01	0.15 ± 0.01	0.067	0.12 ± 0.01	0.14 ± 0.01	0.172	0.12 ± 0.01	0.14 ± 0.01	0.067	0.885
Tetranor PGDM	0.34 ± 0.02	0.35 ± 0.00	0.662	0.33 ± 0.02	0.34 ± 0.00	0.728	0.37 ± 0.02	0.34 ± 0.00	0.049	0.233
PGDM	0.40 ± 0.02	0.41 ± 0.01	0.718	0.40 ± 0.02	0.40 ± 0.01	0.843	0.42 ± 0.01	0.41 ± 0.01	0.756	0.692
Tetranor PGJM	nd	nd	-	nd	nd	-	nd	nd	-	-
Tetranor PGDM lactone	nd	nd	-	nd	nd	-	nd	nd	-	-
**E-Pathway**										
Tetranor PGAM	4.72 ± 2.06	4.77 ± 2.44	0.988	2.35 ± 0.84	7.17 ± 2.82	0.110	9.46 ± 3.03	6.65 ± 3.86	0.570	0.779
Tetranor PGEM	0.25 ± 0.02	0.25 ± 0.01	0.879	0.25 ± 0.01	0.24 ± 0.01	0.806	0.25 ± 0.02	0.25 ± 0.01	0.857	0.704
20-OH-PGE_2_	0.14 ± 0.03	0.11 ±0.01	0.332	0.15 ± 0.02	0.12 ± 0.03	0.106	0.18 ± 0.02	0.11 ± 0.01	0.015	0.743
PGE_2_	nd	nd	-	nd	nd	-	nd	nd	-	-
**F-Pathway**										
Tetranor PGFM	0.06 ± 0.02	0.04 ± 0.01	0.436	0.11 ± 0.03	0.07 ± 0.02	0.186	0.12 ± 0.03	0.05 ± 0.01	0.046	0.276
PGF_2α_	0.10 ± 0.02	0.11 ± 0.02	0.693	0.12 ± 0.02	0.12 ± 0.03	0.920	0.12 ± 0.02	0.10 ± 0.01	0.226	0.530
20-OH-PGF_2α_	1.35 ± 0.36	0.51 ± 0.18	0.045	1.32 ± 0.34	0.57 ± 0.17	0.057	1.75 ± 0.41	0.38 ± 0.14	0.003	0.744
19(*R*)-OH-PGF_2α_	nd	nd	-	nd	nd	-	nd	nd	-	-
**I-Pathway**										
6-*keto*-PGF_1α_	nd	nd	-	nd	nd	-	nd	nd	-	-
**F_2_-IsoPs**										
**15 series**										
2,3-dinor-15-F_2t_-IsoP (2,3-dinor-8-iso-PGF_2α_)	0.83 ± 0.04	0.86 ± 0.01	0.412	0.84 ± 0.04	0.86 ± 0.01	0.572	0.86 ± 0.01	0.86 ± 0.01	0.863	0.990
15-*epi*-15-F_2t_-IsoP (8-iso-15(R)-PGF_2α_)	1.25 ± 0.08	1.60 ± 0.13	0.028	1.25 ± 0.02	1.60 ± 0.08	0.020	1.29 ± 0.07	1.54 ± 0.08	0.028	0.855
15-F_2t_-IsoP (8-iso-PGF_2α_)	0.12 ± 0.01	0.16 ± 0.02	0.089	0.12 ± 0.01	0.15 ± 0.01	0.072	0.12 ± 0.01	0.15 ± 0.01	0.023	0.833
9-*epi*-15-F_2t_-IsoP (8-iso-PGF_2β_)	0.02 ± 0.00	0.02 ± 0.02	0.090	0.02 ± 0.00	0.03 ± 0.03	0.076	0.02 ± 0.00	0.02 ± 0.00	0.582	0.393
15-keto-15-F_2t_-IsoP (8-iso-15-keto-PGF_2α_)	0.91 ± 0.05	1.04 ± 0.09	0.184	0.91 ± 0.05	0.95 ± 0.00	0.319	0.91 ± 0.05	0.95 ± 0.00	0.888	0.339
*ent*-15-*epi*-15-F_2t_-IsoP (*ent*-8-iso-15S-PGF_2α_)	0.57 ± 0.03	0.73 ± 0.12	0.205	0.57 ± 0.03	0.66 ± 0.03	0.052	0.63 ± 0.03	0.62 ± 0.02	0.639	0.388
2,3-dinor-15-*epi*-15-F_2t_	0.69 ± 0.22	0.51 ± 0.15	0.492	0.83 ± 0.23	0.50 ± 0.11	0.201	0.58 ± 0.16	0.58 ± 0.19	0.981	0.907
**5 series**										
5-F_2t_-IsoP	6.51 ± 0.54	8.95 ± 1.16	0.065	6.45 ± 0.72	8.73 ± 0.80	0.040	6.73 ± 0.67	8.08 ± 0.74	0.184	0.776
5-*epi*-5F_2t_-IsoP	1.43 ± 0.08	1.69 ± 0.10	*0.052*	1.40 ± 0.11	1.64 ± 0.06	*0.069*	1.48 ± 0.04	1.60 ± 0.04	*0.057*	0.650
**E2-IsoPs**										
**15 series**										
15-*keto*-15-E_2t_-IsoP (8-iso-15-keto-PGE_2_)	0.81 ± 0.09	0.77 ± 0.07	*0.714*	0.69 ± 0.07	0.78 ± 0.08	*0.380*	0.90 ± 0.11	0.67 ± 0.03	*0.045*	0.395
**Oxylipins from Dihomo-γ-linolenic acid**										
**PGs**										
PGE_1_	0.55 ± 0.03	0.61 ± 0.01	*0.100*	0.53 ± 0.04	0.60 ± 0.01	*0.106*	0.58 ± 0.01	0.59 ± 0.01	*0.142*	0.496
PGD_2_	1.95 ± 0.10	2.08 ± 0.01	*0.197*	1.95 ± 0.10	2.08 ± 0.01	*0.229*	2.05 ± 0.01	2.06 ± 0.01	*0.530*	0.490
PGF_1α_	0.39 ± 0.03	0.39 ± 0.02	*0.967*	0.39 ± 0.03	0.43 ± 0.03	*0.337*	0.45 ± 0.03	0.42 ± 0.03	*0.394*	0.482
**IsoPs**										
15-F_1t_-IsoP (8-iso-PGF_1α_)	0.003 ± 0.000	0.003 ± 0.000	*0.590*	0.003 ± 0.000	0.003 ± 0.000	*0.158*	0.003 ± 0.000	0.003 ± 0.000	*0.491*	0.382
15-E_1t_-IsoP (8-iso-PGE_1_)	3.41 ± 0.59	3.10 ± 0.46	*0.672*	2.70 ± 0.38	3.14 ± 0.53	*0.509*	3.92 ± 0.7	2.40 ± 0.20	*0.045*	0.395

All values are expressed as µM and are reported as mean ± SEM. Legend: 1C: group consuming 1 cup of espresso coffee/day; 3C: group consuming 3 cups of espresso coffee/day; IsoPs: isoprostanes; nd: not detected; PC: group consuming 1 cup of espresso coffee plus 2 cocoa-based products containing coffee twice per day; PGs: prostaglandins. * Effect of time (pre- to post-intervention for each group). Data with *p* < 0.05 are significantly different. ^#^ Effect of treatment (1C vs. 3C vs. PC). Data with *p* < 0.05 are significantly different.

## Data Availability

The data presented in this study are available on request from the corresponding author.

## Supplementary Materials

The following are available online at www.mdpi.com/xxx/s1, Table S1: Analysis of carry over effects for endogenous and oxidatively induced-DNA damage after each treatment. Table S2: Markers of DNA oxidation catabolites in plasma after each treatment. Table S3: Analysis of carry over effects in lipid peroxidation products.
