# Supplementary material for: Effect of Coffee and Cocoa-Based Confectionery Containing Coffee on Markers of DNA Damage and Lipid Peroxidation Products: Results from a Human Intervention Study
Source: Nutrients. 2021 Jul 13;13(7):2399. doi: 10.3390/nu13072399 (PMC8308525; doi:10.3390/nu13072399)
Supplement: Supplementary file 1 [file nutrients-13-02399-s001.zip › nutrients-1258866 - Supplementary version 3.pdf]

nutrients

MDPI

Supplementary Materials

Effect of Coffee and Cocoa-Based Confectionery Containing Coffee on Markers of DNA Damage and Lipid Peroxidation Products: Results from a Human Intervention Study

Daniela Martini [1,†], Raúl Domínguez-Perles [2,†], Alice Rosi [3], Michele Tassotti [3], Donato Angelino [4], Sonia Medina [2], Cristian Ricci [5], Alexandre Guy [6], Camille Oger [6], Letizia Gigliotti [1], Thierry Durand [6], Mirko Marino [1], Hans Gottfried-Genieser [7], Marisa Porrini [1], Monica Antonini [8], Alessandra Dei Cas [8], Riccardo C. Bonadonna [8], Federico Ferreres [9], Francesca Scazzina [3], Furio Brighenti [3], Patrizia Riso [1], Cristian Del Bo' [1,*], Pedro Mena [3,*], Angel Gil-Izquierdo [2,*] and Daniele Del Rio [3]

Table S1. Analysis of carry over effects for endogenous and oxidatively induced-DNA damage after each treatment (n = 21).

Variable	ǀ1C-3Cǀ	ǀ3C-PCǀ	ǀ1C-PCǀ	*p*-value
DNA strand breaks (% DNA in tail, PBS)	5.66 ± 2.61	7.04 ± 4.89	4.26 ± 3.17	0.09
H_2O_2-induced DNA damage (% DNA in tail)	2.88 ± 1.51	4.02 ± 4.61	2.42 ± 3.21	0.21
DNA strand breaks (% DNA in tail, EB)	4.55 ± 3.18	6.12 ± 3.90	5.61 ± 3.70	0.10
FPG-sensitive sites (% DNA in tail)	1.23 ± 1.68	2.11±4.88	3.65 ± 5.71	0.14

Data reported as the absolute value of carry-over effect size ± standard errors. Legend: 1C: group consuming 1 cup of espresso coffee/day; 3C: group consuming 3 cups of espresso coffee/day; PC: group consuming 1 cup of espresso coffee plus 2 cocoa-based products containing coffee twice per day; FPG: formamidopyrimidine DNA glycosylase.

Table S2. Markers of DNA oxidation catabolites in plasma after each treatment (n = 21).

Variable	ǀ1C-3Cǀ	ǀ3C-PCǀ	ǀ1C-PCǀ	*p*-value
8-OH-guanine (nM)	12.37 ± 23.7	3.31 ± 24.28	3.73 ± 14.29	0.32
8-NO2-cGMP(nM)	57.51 ± 74.7	153.30 ± 105.23	18.69 ± 38.60	0.21
8-OH-2′-deoxy-guanosine (nM)	10.98 ± 20.15	1.60 ± 14.5	2.46 ± 7.37	0.19
cGMP (nM)	58.35 ± 58.93	34.19 ± 45.60	6.30 ± 168.12	0.51

Data reported as the absolute value of carry-over effect size ± standard errors. Legend: 1C: group consuming 1 cup of espresso coffee/day; 3C: group consuming 3 cups of espresso coffee/day; PC: group consuming 1 cup of espresso coffee plus 2 cocoa-based products containing coffee twice per day; GMP: guanosine monophosphate.

Table S3. Analysis of carry over effects in lipid peroxidation products (n = 21).

Variable	ǀ1C-3Cǀ	ǀ3C-PCǀ	ǀ1C-PCǀ	*p*-value
**Oxylipins from Arachidonic Acid**				
**PGs**				
**D-Pathway**				
2.3-dinor-11β-PGF$_2$	0.62 ± 0.64	−0.47 ± 0.94	−0.52 ± 0.64	0.84
11-β-PGF2$_\alpha$	0.08 ± 0.05	−0.04 ± 0.06	0 ± 0.07	0.12
Tetranor PGDM	0 ± 0.03	0 ± 0.02	0.01 ± 0.02	0.76
PGDM	0.04 ± 0.03	−0.04 ± 0.05	−0.05 ± 0.05	0.91
Tetranor PGJM	nd	nd	nd	-
Tetranor PGDM lactone	nd	nd	nd	-
**E-Pathway**				
Tetranor PGAM	−8.3 ± 8.03	−9.47 ± 13.95	−11.38 ± 26.21	0.53
Tetranor PGEM	0.04 ± 0.03	−0.04 ± 0.05	−0.04 ± 0.05	0.97
20-OH-PGE$_2$	0.07 ± 0.05	−0.06 ± 0.05	−0.02 ± 0.06	0.78
PGE$_2$	nd	nd	nd	-
**F-Pathway**				
Tetranor PGFM	−0.1 ± 0.12	−0.1 ± 0.09	−0.04 ± 0.09	0.56
PGF$_{2\alpha}$	0.02 ± 0.05	−0.16 ± 0.12	−0.1 ± 0.15	0.16
20-OH-PGF$_{2\alpha}$	0.06 ± 0.08	−0.48 ± 0.86	−0.11 ± 0.87	0.22
19(*R*)-OH-PGF$_{2\alpha}$	nd	nd	nd	-
**I-Pathway**				
6-*keto*-PGF$_{1\alpha}$	nd	nd	nd	-
**F$_2$-IsoPs**				
**15 series**				
2.3-dinor-15-F$_{2t}$-IsoP (2.3-dinor-8-iso-PGF$_{2\alpha}$)	0.01 ± 0.03	−0.01 ± 0.04	−0.02 ± 0.04	0.88
15-*epi*-15-F$_{2t}$-IsoP (8-iso-15(R)-PGF$_{2\alpha}$)	0.71 ± 0.3	−0.5 ± 0.56	−0.4 ± 0.48	0.43
15-F$_{2t}$-IsoP (8-iso-PGF$_{2\alpha}$)	0.07 ± 0.05	−0.01 ± 0.08	−0.03 ± 0.07	0.31
9-*epi*-15-F$_{2t}$-IsoP (8-iso-PGF$_{2\beta}$)	0.002 ± 0.002	−0.01 ± 0	0 ± 0.01	0.43
15-keto-15-F$_{2t}$-IsoP (8-iso-15-keto-PGF$_{2\alpha}$)	0.01 ± 0.01	−0.4 ± 0.44	0 ± 0.02	0.55
ent-15-*epi*-15-F$_{2t}$-IsoP (*ent*-8-iso-15S-PGF$_{2\alpha}$)	0.06 ± 0.08	−0.04 ± 0.44	−0.11 ± 0.12	0.67
2.3-dinor-15-*epi*-15-F$_{2t}$	0.68 ± 0.69	−0.57 ± 1.09	−0.59 ± 0.73	0.56
**5 series**				
5-F$_{2t}$-IsoP	5.49 ± 2.48	−5.35 ± 5.87	−4.69±4.59	0.81
5-*epi*-5F$_{2t}$-IsoP	0.34 ± 0.27	−0.08 ± 0.46	−0.27±0.36	0.44
**E2-IsoPs**				
**15 series**				
15-*keto*-15-E$_{2t}$-IsoP (8-iso-15-keto-PGE$_2$)	0.52 ± 0.52	0.03 ± 0.28	0.31 ± 0.48	0.32
**Oxylipins from Dihomo-γ-linolenic acid**				
**PGs**				
PGE$_1$	0.05 ± 0.04	−0.01 ± 0.06	−0.03 ± 0.05	0.91
PGD$_2$	0.06 ± 0.09	−0.07 ± 0.04	−0.03 ± 0.12	0.92
PGF$_{1\alpha}$	0.1 ± 0.13	−0.02 ± 0.11	0.03 ± 0.14	0.33
**IsoPs**				
15-F$_{1t}$-IsoP (8-iso-PGF$_{1\alpha}$)	0.002 ± 0.002	−0.001 ± 0.001	0 ± 0.002	0.72
15-E$_{1t}$-IsoP (8-iso-PGE$_1$)	3.43 ± 3.41	0.18 ± 1.83	2.04 ± 3.16	0.54

Data reported as the absolute value of carry-over effect size ± standard errors. Legend: 1C: group consuming 1 cup of espresso coffee/day; 3C: group consuming 3 cups of espresso coffee/day; PC: group consuming 1 cup of espresso coffee plus 2 cocoa-based products containing coffee twice per day; E2-IsoPs 15-series: total E2-isoprostanes 15-series; F2-IsoPs 15 series: total F2-isoprostanes 15 series; F2-IsoPs 5 series: total F2-isoprostanes 5 series; IsoPs: isoprostanes; PG: prosta-

glandins; PGs D-Pathway: total prostaglandins D-pathway; PGs E-Pathway: total prostaglandins E-pathway; PGs F-Pathway: total prostaglandins F-pathway; PGs I-Pathway: total prostaglandins I-pathway; IsoPs from DGLA: total isoprostanes from dihomo-γ-linolenic acid; PGs from DGLA: prostaglandins from dihomo-γ-linolenic acid; NS: not significant.
